# Lip Enhancement and Perioral Area Correction With New Hyaluronic Acid Dermal Fillers: A Prospective Safety Study

**DOI:** 10.1093/asjof/ojaf101

**Published:** 2025-08-29

**Authors:** Sophie Converset, Isaac Bodokh, Henry Delmar, Elisabeth Domergue Than Trong, Cécile Winter, Philippe Kestemont

## Abstract

**Background:**

Injectable hyaluronic acid (HA) is the gold standard for the aesthetic treatment of facial volume and wrinkles.

**Objectives:**

The authors of this study aim to evaluate the safety and performance of 2 new HA fillers for the treatment of the lips and perioral area.

**Methods:**

This multicenter prospective study enrolled 72 patients. All patients received lip enhancement with ESTYME LIPS (EST_LP_, SYMATESE, Chaponost, France), and 61 of them also had correction for lip fine lines and nasolabial folds (NLFs) with ESTYME SMOOTH (EST_SM_). Assessments were conducted by injector-investigators, the patients, and an independent evaluator. The primary endpoint was the proportion of patients with at least 1 injection-site reaction (ISR) lasting beyond 14 days. Secondary endpoints included adverse events, aesthetic improvements, lip functionality, and participant satisfaction, assessed over 12 months.

**Results:**

After 30 days, at least 1 ISR was observed in 35% of the participants on the lips, 2% on the fine lines, and 2% on the NLF. At 12 months, an improvement of at least 1 point was still observed in 66% of participants on the lips, 74% on the fine lines, and 54% on the NLFs. Throughout the study, lip functionality was not affected by their treatment and both assessors and patients rated overall aesthetic improvement and good satisfaction.

**Conclusions:**

In this limited cohort, EST_LP_ and EST_SM_ proved to be safe and effective options for lip enhancement as well as correction of NLFs and lip fine lines, demonstrating sustained results in the majority of patients for up to 12 months.

**Level of Evidence:**

4 (Therapeutic)
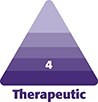

The face is one of the body areas where the initial signs of skin aging prominently appear. Age-related changes in the face are intrinsic, stemming from genetic factors, and exacerbated by extrinsic factors, such as solar radiation (photoaging), cigarette smoking, and pollutants.^1,2^

Over time, the skin gradually becomes thinner and drier, leading to the appearance of wrinkles and surface irregularities. The loss of surface area of the dermal–epidermal interface contributes to increased skin fragility and reduced nutrient transfer between the dermis and the epidermis. Histologically, the dermis undergoes atrophy, characterized by a reduced number of fibroblasts, a decline in subdermal adipose tissue, and a simultaneous decrease in collagen.^3^

The lips and perioral tissues represent a face site that is particularly susceptible to manifest signs of aging. During puberty, the lips are fleshy and full owing to the hypertrophy of the orbicularis oris muscle and glandular components. However, with the action of time, these features gradually diminish, leading to a loss of definition and a flattening of the lips. The upper lip may elongate, altering the natural curvature of the cupid's bow. Simultaneously, lip fine lines (known as perioral lines or perioral rhytids) develop vertically around the vermillion border, and nasolabial folds (NLFs) become apparent.^4^ The loss of structural support, which enhances the prominence of wrinkles and NLFs, can induce changes in the perioral region, altering the perception of facial expressions in aging individuals. This area has, therefore, a major influence on the age perception and judgments of facial attractiveness in people.^5^

Patients seeking facial rejuvenation or lip augmentation commonly manifest concerns about the aging appearance of the perioral region and lip thinning. Associated with attractiveness, lip fullness has become increasingly popular over the past years.^6-8^ Responding to their request often includes lip reshaping to address volume loss and correction of lip fine lines and NLFs to rejuvenate imperfections around the lips.

Injectable hyaluronic acid (HA) has emerged as the gold standard for treating facial volume loss and wrinkles. Renowned for their natural results without invasive procedures, HA fillers have nearly displaced collagen fillers, owing to their prolonged clinical efficacy (6-12 months compared with 2-4 months), absence of skin testing requirements, reduced allergic side effects, and enhanced pliability.^9^

The purpose of HA injections is 2-fold: to increase and control tissue water content for natural volumizing to lift creased tissues to smoothen the skin, thereby achieving rejuvenation across various facial areas.^9^

With the rising demand for facial aesthetic interventions, manufacturers have expanded treatment indications, developing a comprehensive range of HA fillers for facial rejuvenation and volumization. These fillers target areas such as NLFs, the midface, periorbital regions, lips, and perioral applications. Each product is defined by its rheological properties, which determine its behavior. These characteristics are primarily influenced by the unique crosslinking technology of each filler line.^9,10^

In this context, a new generation of HA fillers has been developed (SYMATESE, Chaponost, France) using a patented cold cross-linking process. This technology is referred to as “COLD-X TECHNOLOGY by Symatese” in Europe and the United States, and “CXLD Technology by Symatese” in China. Each product of this range is designed for a specific facial area, allowing for a more tailored approach to treatment. The product range is named “ESTYME” in Europe, “EVOLYSSE” in the United States, and “PRECISE” in China. This study focuses on 2 distinct products: ESTYME LIPS (EST_LP_) for lip volume and shape and ESTYME SMOOTH (EST_SM_) for perioral area correction, including lip fine lines and NLFs. The objective of the authors of this study is to evaluate the safety and aesthetic effectiveness of these products over a 12-month period.

## METHODS

### Ethical Approval

This study was evaluated by a French Independent Ethics Committee, the “Comité de Protection des Personnes NORD-OUEST I,” and was registered at the Agence Nationale de Sécurité du médicament et des produits de Santé (no. 2020-A01099-30). The clinical investigation was conducted in accordance with the French law in force, according to the Declaration of Helsinki (1964) and guidelines for good clinical practice (ISO 14155:2012), Medical Device Regulation (EU) 2017/745 of April 5, 2017, and the EU General Data Protection Regulation 2016/769 and in compliance with the approved Clinical Investigation Plan. Before study entry, all patients were fully informed verbally and in writing about the nature and aim of the clinical investigation and gave their written informed consent.

### Study Design

This is an interventional, multicenter, prospective study evaluating the safety and efficacy of 2 HA fillers. EST_LP_ is used to correct the shape and volume of the lips (upper and lower), whereas EST_SM_ is used to correct lip fine lines (upper and lower) and NLFs. To ensure a comprehensive safety assessment, most patients received injections in multiple facial areas, representing a higher-risk scenario that closely mirrors real-world clinical practice.

These investigational products are sterile, bioabsorbable, and colorless soft-tissue fillers from the ESTYME range, designed to modify the skin anatomy and facial appearance for aesthetic purposes.

Both products are composed of 20 mg/mL of HA gel and 0.3% lidocaine and supplied in a 1.0 mL prefilled single-use syringe but differ in terms of viscoelastic properties and cohesiveness. To ensure efficient lip enhancement without uncontrolled swelling, EST_LP_ is moderately crosslinked (elastic modulus [*G*′] of 227 Pa), cohesive, and hydrophilic, whereas EST_SM_ is the least crosslinked (*G*′ of 122 Pa) in the range, offering more superficial filling and a smoothing effect. The *G*′ values are the average measurements from multiple batches.

The study was conducted in France in 3 investigational centers over a 12-month period with visits at Day 0 (D0, injection day), Day 14 (D14), Day 30 (D30), 3 months (M3), 6 months (M6), 9 months (M9), and 12 months (M12).

### Interventions

Depending on the desired effect, patients received either EST_LP_ for lip enhancement only or EST_LP_ and EST_SM_ for lip enhancement (volume and shape) and perioral area correction, including lip fine lines and NLFs. The injection techniques and treatment volume were left to the discretion of the investigators. It was recommended that no more than 6 mL of product be used per patient in a single session without touch-up (2 mL of EST_LP_ for both lips and 4 mL of EST_SM_ for both NLF and lip fine lines), although this amount could vary. The injections were performed at 3 sites by 5 investigators, all of whom were qualified healthcare practitioners experienced in the use of injectable HA (dermatologists or aesthetic surgeons).

### Participants

The study enrolled healthy adults seeking an aesthetic procedure to correct volume and shaping of the lips, with the option for those interested to also rejuvenate the perioral area.

Patients with upper and/or lower lips ranging from very thin to moderately thin were included in this study. In terms of the perioral area, patients with apparent lip fine lines and NLFs were eligible for treatment. Pregnant or nursing women were not included in the study. In addition, patients suffering from uncontrolled disease or pathology (such as diabetes, autoimmune pathology, cardiac pathologies, hepatic deficiency, epilepsy, and porphyria) as well as those with cutaneous disorders, inflammation, or infection were excluded. Other main exclusion criteria included patients who underwent correction in the studied areas using nonpermanent fillers or neurotoxins within 12 months earlier, as well as those who received a permanent filler; patients with a known allergy or hypersensitivity to HA or lidocaine; those with a known bleeding disorder or receiving medication likely to increase the risk of bleeding; and patients who received chemotherapy agents, immunosuppressive medications, or systemic corticosteroids within the past 3 months.

### Assessments

Local tolerance was evaluated based on the presence and severity (mild, moderate, or severe) of any injection-site reactions (ISRs). These reactions were assessed by the injector-investigators during each follow-up visit and by the patients through a daily diary for 30 days following the injection. The monitored ISRs included redness, pain/tenderness, induration/firmness, swelling, lumps/bumps, bruising, itching, and discoloration. General safety was assessed by the injector-investigators through reporting adverse events (AEs) and adverse device effects (ADEs) at each time point. ISRs that appeared after 30 days, lasted more than 30 days, or required corrective treatment were also reported as ADEs.

Furthermore, the injector-investigators specifically evaluated lip functionality by assessing the following parameters: texture, firmness, movement, function, and sensation, described as normal or abnormal.

Performance assessments were conducted at various time points in person by injector-investigators and patients and on photographs by an independent evaluator. The independent assessor, an aesthetic surgeon experienced in the use of injectable HA, was aware that the patients had received lip treatment but was unaware whether they had received additional treatment for the NLFs or perioral lines. The performance was evaluated area by area thanks to dedicated scales. Lips, lip fine lines, and NLFs were assessed distinctly by the injector-investigators in person at D0 (before injection), D30, M3, M6, M9, and M12, as well as by the independent investigator on standardized photographs taken on D0 (before injection), D30, M6, and M12. The injector-investigators and the independent evaluator used the same assessment scales, having received previous training to ensure consistency in their application throughout the study. The lips (upper and lower) were evaluated for volume and shape using a 5-point scale, ranging from very thin to plump, similar to validated scales commonly used in clinical practice for this area (Supplemental Table 1).^11-13^ Although lip fine lines and NLFs were assessed using the 6-point validated Lemperle rating scale, which categorizes wrinkle severity from no wrinkle to very deep wrinkle (Supplemental Table 2).^14^ Overall aesthetic improvement was evaluated in person by the injector-investigators and the patients themselves at D30, M3, M6, M9, and M12, using the Global Aesthetic Improvement Scale (GAIS; Supplemental Table 3).

Immediately after the injection in each treated area, patients rated their pain level using a visual analog scale anchored by “no pain” (0) to “worst imaginable pain” (100) on a 100 mm scale. Lastly, at each follow-up visit (D30-M12), patients completed questionnaires to assess their satisfaction and quality of life (QoL) related to the facial treatment.

### Measures and Endpoints

#### Safety Endpoints

The primary objective of this study was to evaluate the safety of EST_LP_ for lip enhancement and EST_SM_ for lip fine lines and NLF treatments 30 days after injection. This was assessed for each treated area by the proportion of patients with at least 1 ISR lasting beyond 14 days after injection, as reported by the patient between D15 and D30, and by the injector-investigator on D30.

Secondary safety endpoints were (1) ISRs assessed by patients from D1 to D30 and by injector-investigators at each follow-up visit, (2) global safety related to the study (AE/ADE) recorded throughout the 12-month follow-up period by injector-investigators, and (3) safety parameters of lips assessed by injector-investigators on D0 (baseline) and at each follow-up visit.

#### Efficacy Endpoints

The efficacy of EST_LP_ and EST_SM_ was a secondary objective of the study. The main efficacy endpoint was the proportion of patients showing an improvement of at least 1 point from the baseline in lip volume, severity of lip fine lines, and severity of NLFs, as assessed by the injector-investigators using the specific scale to the treated area on D30. The other secondary efficacy endpoints included (1) the proportion of patients showing an improvement of at least 1 point from the baseline in each treated area, measured at other time points by the injector-investigators and an independent evaluator; (2) global aesthetic improvement of the face, defined as the proportion of patients who had an overall improvement of at least 1 point in facial appearance compared with baseline; (3) patients’ satisfaction and QoL; and (4) pain reported by patients immediately after injection.

### Statistical Analysis

Primary and secondary endpoint analyses were performed on both the intention to treat (ITT; all patients who received at least 1 injection of medical devices) and the per protocol (PP; all patients from the ITT population without any major deviation from the protocol) populations. Baseline description and safety analyses were realized on the ITT population.

Quantitative variables were summarized using descriptive statistics (mean, median, standard deviation, first and third quartiles, minimum and maximum values), and CIs (using the Student's law), where applicable. Qualitative variables were described by the frequency and percentage of each category, along with CIs (using the Wilson score method or binomial method). No statistical tests were performed for this clinical investigation.

The primary endpoint analysis focuses on the proportion of patients with at least 1 ISR beyond 14 days after injection, with criteria assessed for each area. The proportion is presented with a 95% CI. The main efficacy endpoint analysis assesses the proportion of patients with at least a 1-grade improvement on dedicated scales for each treated area 30 days after injection, presented with the 95% CI.

## RESULTS

### Patient Disposition

In total, 73 patients were enrolled in this study. Of those, 1 patient withdrew his consent before injection, and, therefore, a total of 72 patients were included in the ITT population. One other patient was excluded from the study because of a major deviation (wrong product injected); thus, 71 patients finally constituted the PP population.

The patients were predominantly females (67 women, 93%), with only 5 males (7%). The overall mean age was 55.7 years (range, 34-75 years). A total of 11 (15%) patients received EST_LP_ in the lips only (lips only), whereas 61 (85%) patients were injected with both EST_LP_ in the lips and EST_SM_ in the perioral area (lips, lip fine lines, and NLFs). Patients were further stratified into 3 subgroups by injected area for analyses: the lip subgroup (*n* = 72), the lip fine lines subgroup (*n* = 61), and the NLFs subgroup (*n* = 61). Demographic data are presented in Table 1.

**Table 1.** Patient Demographics, Intention-to-Treat Population (*n* = 72)

Variables	Value
Age, years	
Mean (SD)	56 (9.5)
Range	34-75
Sex, ***n*** (%)	
Male	5 (7)
Female	67 (93)
Treatment area, ***n*** (%)	
Lips	72 (100)
Lips only	11 (15)
Lips, lips fine lines, and NLFs	61 (85)
Subgroups for analyses, ***n*** (%)	
Lip subgroup	72 (100)
Lip fine lines subgroup	61 (85)
NLF subgroup	61 (85)

*n*, number of patients; NLF, nasolabial fold; SD, standard deviation.

On average, 0.5 mL of EST_LP_ was injected per lip in a single session with a maximum injected volume of 1.0 mL for the upper lip and 1.3 mL for the lower lip. An average of 0.5 and 0.6 mL of EST_SM_ was injected into the perioral lines and each NLF, respectively, also in a single session. The maximum injected volume was 1.4 mL for the lip fine lines and 1.2 mL for each NLF.

### Safety Outcomes

#### Injection-Site Reactions

ISRs were evaluated by the patients themselves from the day of injection (D0) to D30 and by the injector-investigators throughout the study up to the M12 visit. Overall, a higher proportion of ISRs persisting beyond 14 days were noted in patients injected in the lips, compared with those injected in the perioral area.

Based on patients’ self-assessment between Day 15 and Day 30 post injection, 72% (95% CI, 58.4-82.2) reported experiencing an ISR in the lips, 23% (95% CI, 11.7-39.7) in the lip fine lines, and 34% (95% CI, 20.6-50.7) in the NLFs. At the Day 30 evaluation, investigators reported that 35% (95% CI, 24.1-46.9) of patients exhibited at least 1 ISR in the lips, whereas only 2 patients (3%, 95% CI, 0.6-12.4) had ISRs in the perioral area, 1 affecting the lip fine lines (2%, 95% CI, 0.1-10.0) and the other patient in the NLFs (2%, 95% CI, 0.1-10.0).

**Figure 1.**
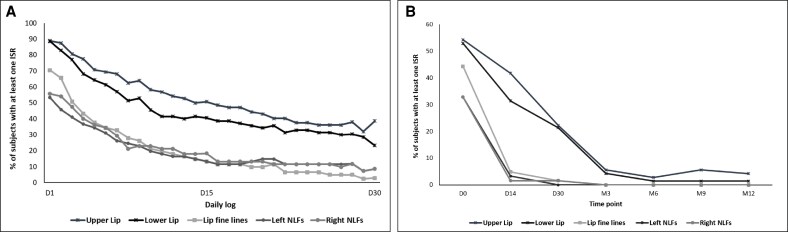
Patients with at least 1 injection-site reaction in the intention-to-treat population (*n* = 72) throughout the study period (A) from Day 1 to Day 30, assessment by patients and (B) from Day 0 (after injection) to Month 12, assessment by injector-investigators.

Looking specifically at the injection areas, the proportion of patients with at least 1 ISR in each area decreased over time, as reported by both patients (Figure 1A) and investigators (Figure 1B), with most ISRs occurring between Day 0 and Day 30.

**Figure 2.**
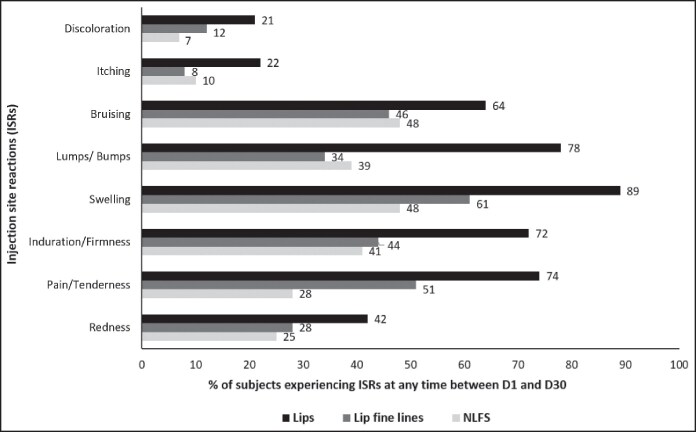
Percentage of patients experiencing ISRs at any time between Day 1 and Day 30, as assessed by patients in the intention-to-treat population (*n* = 72). The patients documented the occurrence of predefined injection-site reactions daily from Day 1 to Day 30 in a diary.

**Figure 3.**
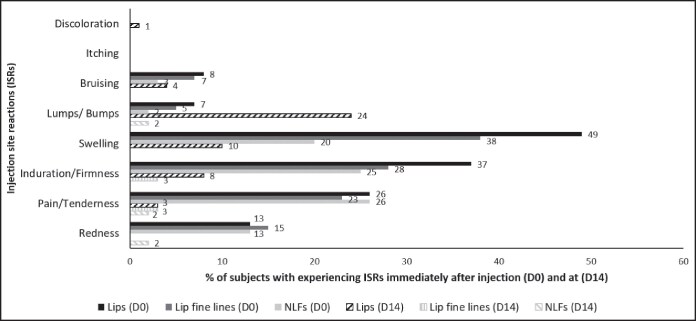
Percentage of patients experiencing injection-site reactions (ISRs) immediately after injection (Day 0) and at Day 14, as assessed by injector-investigators in the intention-to-treat population (*n* = 72). The injector-investigators recorded the occurrence of predefined ISRs at each follow-up visit, with the highest incidence observed at Day 0 after injection and Day 14.

The majority of ISRs were mild in intensity, whether assessed by investigators or reported by patients. In the lips and lip fine lines, swelling was the most commonly reported ISR. For the NLFs, patients most frequently reported swelling and bruising, whereas investigators most commonly observed pain/tenderness (Figures 2, 3).

#### Adverse Events, Adverse Device Effects, and Device Deficiencies

Overall, 35 patients reported 117 AEs (49%, 95% CI, 36.8-60.6), of which 1 was considered as a serious AE not related to the device nor the intervention (loss of consciousness and craniocerebral injury).

In total, 18 patients (25%, 95% CI, 15.9-36.8) experienced at least 1 AE related to the studied devices—ADEs (Table 2). The most common events were lumps (14%) and indurations (8%) on the lips. The number of ADEs was 31, and nearly all of them (97%) were mild in severity. Most ADEs resolved spontaneously without sequelae. Except for cases where the product was felt in the upper lip and headache, which required massage and medication, respectively, no actions were taken to treat ADEs. At the end of the study, 5 mild ADEs remained unchanged on the lips (4 cases of induration and 1 of lump formation), whereas none were reported in the perioral areas. There was no major technical issue related to the devices during their use.

**Table 2.** Adverse Device Effects in the ITT Population (*n* = 72)

ITT population, *n* = 72					
	*n* (%)	Severity	SAE	Relation to device	Relation to intervention
ADEs	18 (25)				
Lumps in lip	10 (14)	Mild^a^	No	Likely	Not linked
Induration of lip	6 (8)	Mild	No	Likely	Not linked
Lip injection-site induration	2 (3)	Mild	No	Not linked	Likely
Lip injection-site nodule	2 (3)	Mild	No	Not linked	Likely
Lip injection-site pain	1 (1)	Mild	No	Not linked	Likely
Perioral lines injection-site induration	1 (1)	Mild	No	Not linked	Likely
Product felt in upper lip (oral)	1 (1)	Mild	No	Certain	Certain
Headache	1 (1)	Mild	No	Unlikely	Unlikely

ADE, adverse device effect; *n*, number of patients; ITT, intention to treat; SAE, serious adverse event. ^a^Moderate for 1 patient.

#### Lip Functionality

Lip parameters (texture, firmness, movement, function, and sensation) were assessed by investigators before and after injection. Treatment did not compromise lip sensation, sensitivity, or function and did not have an impact on speech articulation. Specifically, on D30, lip parameters were reported as normal for 99% of patients; 1 patient reported a slightly abnormal texture of the upper lip. From Month 3 to Month 12, lip parameters were reported as normal for all patients (100%).

### Efficacy Outcomes

#### Patients With at Least 1 Grade Improvement on Each Treated Area

**Figure 4.**
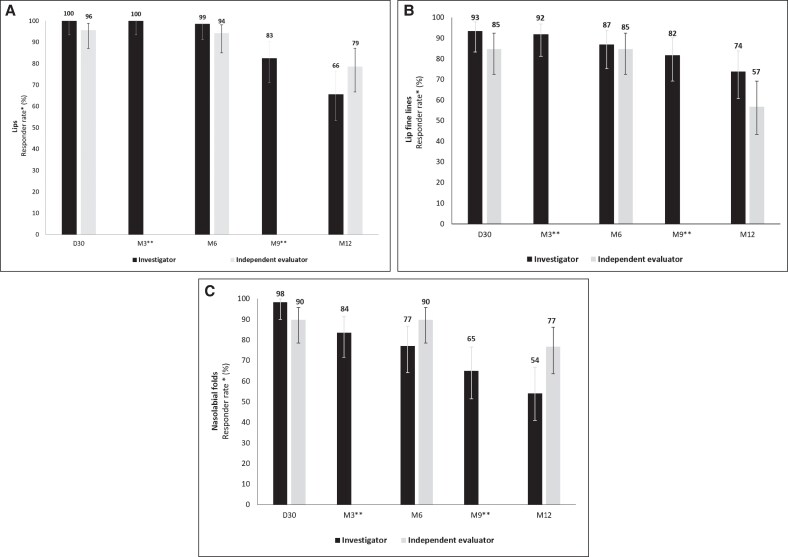
Responder rates by treated area in the intention-to-treat population (*n* = 72). The injector-investigators and the independent evaluators assessed the following: (A) lip volume (upper and lower) using a 5-point scale ranging from very thin to plump; (B) lip fine lines and (C) nasolabial fold severity using the 6-point Lemperle rating scale, which ranges from no wrinkles to very deep wrinkle. *The responder rate was defined as the percentage of patients who had at least a 1-point improvement from baseline in the concerned area. **Assessments were not performed by the independent evaluator at Month 3 and Month 9. Error bars indicate 95% CI.

Following the injection of EST_LP_, all patients (100%) demonstrated a ≥1-point improvement on the lip volume scale as assessed by injector-investigators on D30. This enhancement persisted at 3 months (100%), 6 months (99%), 9 months (83%), and 12 months (66%). Based on the patients’ photographs, the independent evaluator observed a similar sustained enhancement throughout the study, with a higher rate of improvement at 12 months (79%). Despite this difference, the 95% CIs for the percentages of patients considered improved by both the injector-investigators and the independent evaluator overlapped, suggesting no statistically significant difference between the 2 assessments (Figure 4A).

In relation to the perioral areas, injector-investigators reported a ≥1-point improvement in the severity of lip fine lines in 93% of the patients 30 days post injection of EST_SM_. Improvement was maintained over 12 months (74%). Similarly, for NLF treated with EST_SM_, injector-investigators recorded a decrease in NLF severity in 98% of patients. At M12, 54% of patients still demonstrated improvement. The independent evaluator also reported consistent improvement over time, with some notable differences. At 30 days, improvement rates for lip fine lines and NLFs were lower (85% and 90%, respectively) compared with the injector-investigators’ in-person assessments (93% and 98%, respectively). At 12 months, the independent evaluator recorded a lower improvement rate for lip fine lines (57%) but a higher rate for NLFs (77%). Despite these differences, the 95% CIs for the percentages of patients considered improved by both the injector-investigators and the independent evaluator also overlapped in the perioral areas, suggesting no statistically significant difference between the 2 assessments (Figure 4B, C).

#### Global Aesthetic Improvement

**Figure 5.**
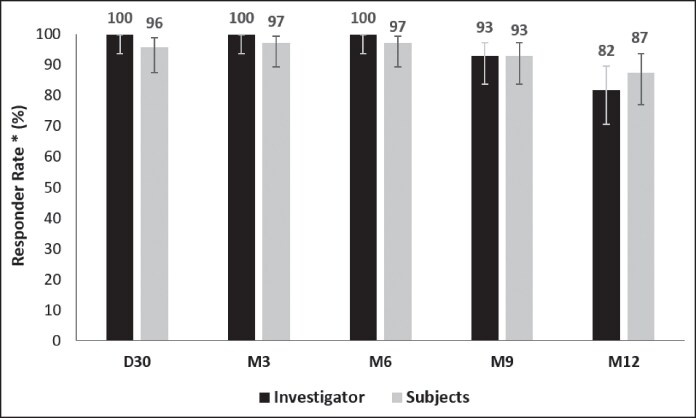
Investigator- and patient-assessed aesthetic improvement (Global Aesthetic Improvement Scale, GAIS) in the intention-to-treat population (*n* = 72). Overall aesthetic improvement was evaluated with the GAIS on a 5-point scale from very much improved (1), much improved (2), improved (3), no change (4), to worse (5). The responder rate was defined as the percentage of patients who were assessed as either “very well improved,” “well improved,” or “improved” on the GAIS. Error bars indicate 95% CI.

The global aesthetic improvement of the face was evaluated by both injector-investigators and patients after 30 days and at each subsequent follow-up time using the GAIS. On D30, investigators noted an improvement in all patients, whereas 96% of the patients self-reported an improvement. Improvement was sustained up to 12 months after injection, with investigators observing improvement in 82% of cases, slightly lower than the 87% reported by patients. The presentation of the results with 95% CIs does not suggest a significant difference between the assessments of the injector-investigators and those of the patients (Figure 5).

Regarding the patients’ self-assessments, 3 (4%) reported no improvement on D30, 2 (3%) at M3 and M6, 5 (7%) at M9, and 9 (12%) at M12. The investigators reported that 5 patients (7%) showed no improvement at M9 and 13 (18%) at M12. It is important to highlight that no patient was reported as having worsened, according to either the investigators’ evaluations or the patients’ self-assessments.

### Patient Satisfaction and Quality of Life

**Figure 6.**
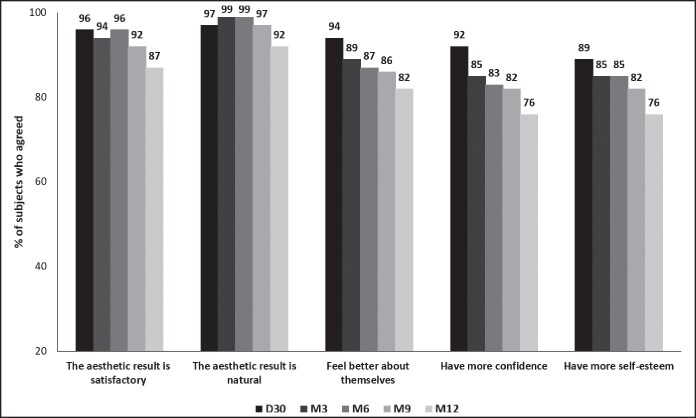
Patients assessed satisfaction and improvement of quality of life in the intention-to-treat population (*n* = 72). Patients completed a questionnaire on satisfaction, feeling better, confidence, and self-esteem.

Patients responded to a questionnaire to assess their overall satisfaction and QoL up to 12 months after injection. Most of the patients (87%) were satisfied with the results of EST_LP_ and EST_SM_, considering the aesthetic result satisfactory and natural. Overall, 76% of patients reported an improvement in their QoL throughout the study, including feeling better about themselves, having more self-esteem and gaining confidence (Figure 6).

**Figure 7.**
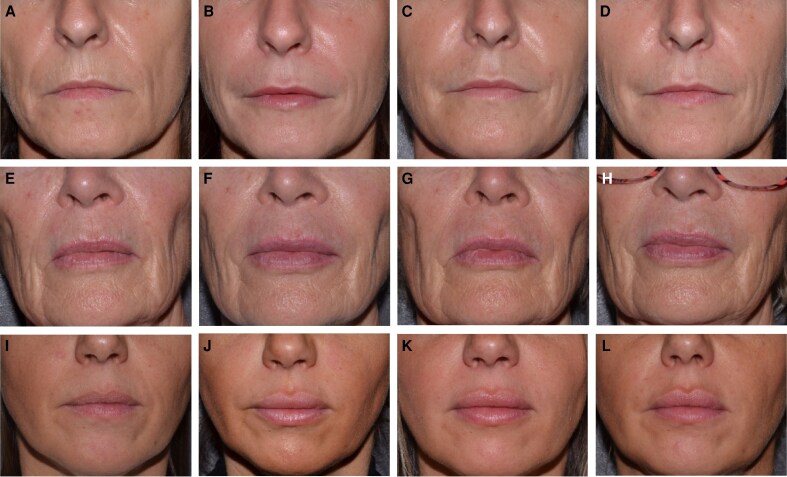
Photographs of representative patients at baseline, 30 days, 6 months, and 12 months after treatment. (A-D) A 51-year-old woman treated with EST_LP_ for the lips (upper lip: 0.6 mL; lower lip: 0.45 mL) and with EST_SM_ for lip fine lines (0.4 mL) and nasolabial folds (0.5 mL for each side) at (A) baseline, (B) 30 days, (C) 6 months, and (D) 12 months after treatment. (E-H) A 65-year-old woman who received 0.3 mL of EST_LP_ for each lip, and EST_SM_ for lip fine lines (0.6 mL) and nasolabial folds (0.7 mL for the right side, 0.9 mL for the left side) at (E) baseline, (F) 30 days, (G) 6 months, and (H) 12 months after treatment. (I-L) A 43-year-old woman treated with EST_LP_ for the lips (upper lip: 0.6 mL; lower lip: 0.35 mL) and with EST_SM_ for lip fine lines (0.2 mL) and nasolabial folds (0.8 mL for each side) at (I) baseline, (J) 30 days, (K) 6 months, and (L) 12 months after treatment.

Figure 7 shows photographs of representative patients before, 30 days, 6 months, and 12 months after treatment for lips, lip fine lines, and NLFs.

### Pain

All patients reported mild pain following injection of EST_LP_ (mean pain score: 21.3 mm ± 17.5) and EST_SM_ (mean pain scores of 18.9 mm ± 17.6 for lip fine lines and 14.5 mm ± 12.3 for NLFs) based on a 100 mm measurement scale.

## DISCUSSION

HA dermal fillers are versatile agents for facial rejuvenation, with various products available based on different physical properties, allowing the selection of the most adapted product for the specific indication, the severity of the defect, and the expected results of each patient.^15,16^

Performing the crosslinking process at a low temperature helps preserve the integrity of the long HA chains and reduces the amount of BDDE (1,4-butanediol diglycidyl ether) crosslinking agent needed to achieve the desired gel properties. Minimizing HA chain fragmentation is intended to enhance the safety, performance, and longevity of the fillers. Based on this cold crosslinking approach, a new range of HA fillers has been developed for different areas in full-face rejuvenation, each designed with specific physical properties to deliver optimal results. The first comparative clinical study of one of these products demonstrated excellent safety and performance outcomes compared with a commonly used filler, thereby clinically validating the innovative technology.^17^ The present study aimed to evaluate the safety and efficacy of 2 new HA-based fillers, EST_LP_ for lip enhancement and EST_SM_ for correction of the perioral area that includes lip fine lines and NLFs. Most patients underwent injections with both products in 3 distinct areas to comprehensively address lip volume loss and surrounding wrinkles, while ensuring a safety assessment that mirrors real-world clinical practice.

Safety was the primary objective of the study, and the patients generally tolerated both products well. Most reported ISRs were as expected for HA-based soft-tissue filler injections, including swelling and firmness, lasting 2 weeks or less and resolving without complications.

A higher proportion of ISRs persisting beyond 14 days occurred in patients injected in the lips compared with perioral areas. According to the investigators, 35% of patients reported at least 1 ISR lasting beyond 14 days following EST_LP_ injections into lips. EST_SM_ showed lower ISR rates; only 2 patients experienced an ISR in the perioral areas. These results are consistent with previous studies reporting the incidence of ISR beyond 2 weeks following the injection of HA fillers, where the proportion of patients experiencing any ISR ranged between 95% and 98%.^18-20^

The proportion of AEs and ADEs fell within the expected range for fillers. Across studies, ∼10% to 52% of patients experienced an ADE.^18,19^

Similarly, in a systematic review of published literature conducted by Trinh et al, 23 included articles evaluating 28 different fillers reported a 30% incidence of AEs, with 466 events among 1545 treated patients.^21^

Overall, patients only reported mild pain after injections, likely because of the presence of lidocaine, an anesthetic commonly used in dermal fillers to reduce pain.^22,23^ Lip injections for volume enhancement and shape improvement did not affect lip sensation, sensitivity, or function, nor did they affect diction or pronunciation, consistent with initial measurements showing normal lip-specific functionalities and sensitivity.^24,25^

Each treated area showed high success rates and meaningful improvements according to the assessments of the injector-investigators and the independent evaluator, both in the short and long term. According to assessments made in person by the injector-investigators, response rates were notably high, with 100% of patients achieving a ≥1-point improvement on the lip volume scale 30 days following the injection of EST_LP_ into the lips and, respectively, 93% and 98% of patients achieving a ≥1-point improvement on the wrinkle severity scale after injection of EST_SM_ into the lip fine lines and the NLFs. Based on photographs, the independent evaluator reported slightly lower improvement rates at 30 days; however, the results remained satisfactory. On the GAIS, injector-investigators noted an improvement in all patients after 30 days, whereas 96% of the patients self-reported an improvement.

Positive outcomes were sustained over the long term with both products. Up to 12 months after injection, 66% of patients injected with EST_LP_ still had an improvement of at least 1 point from the baseline in lip volume. These findings exceed those previously reported by Czumbel et al in a meta-analysis on HA-based dermal fillers for lip augmentation, which revealed that 46% of patients had increased lip fullness by 1 point or more 12 months after a single HA injection.^26^

Similarly, for patients injected with EST_SM_, 74% of patients injected in the lip fine lines and 54% of those treated in the NLFs still noted an improvement of at least 1 point from the baseline in wrinkle severity 12 months after injection, based on in-person assessments, outperforming other HA fillers used in the lip fine lines and displaying similar efficacy to other HA fillers used in the NLFs.^18,19,24,27^

Both patients and investigators were satisfied with the overall aesthetic appearance of the face, with high GAIS responder rates after 12 months. Patients were satisfied with the aesthetic results and also reported improvements in their QoL, as well as increased self-perception, confidence, and self-esteem.

This study has several limitations, including its open-label design, lack of a control group, limited cohort size, and the use of a partially blinded evaluation, all of which may reduce the strength and generalizability of the findings. Larger, well-controlled studies are needed to establish more definitive conclusions.

This study, highlighting the safety and performance of EST_LP_ and EST_SM_, has significantly contributed to their authorization in Europe for lip correction with EST_LP_ and for the correction of NLFs and lip fine lines with EST_SM_. At the time of writing, EST_LP_ has not yet received approval in other territories, whereas EST_SM_ has been FDA approved under the name EVOLYSSE SMOOTH.

## CONCLUSIONS

This study assessed the safety and performance of 2 new HA fillers: EST_LP_ for lip enhancement and EST_SM_ for correction of the perioral area. Despite the limited cohort size, the results suggest that EST_LP_ and EST_SM_ may represent valuable options for lip augmentation and perioral rejuvenation within a comprehensive aesthetic treatment plan.

## Supplemental Material

This article contains supplemental material located online at https://doi.org/10.1093/asjof/ojaf101.

## Supplementary Material

ojaf101_Supplementary_Data

## References

[1] Rexbye H, Petersen I, Johansen M, Klitkou L, Jeune B, Christensen K. Influence of environmental factors on facial ageing. Age Ageing. 2006;35:110–115. doi: 10.1093/ageing/afj031 16407433

[2] Farage MA, Miller KW, Elsner P, Maibach HI, Farage MA. Intrinsic and extrinsic factors in skin ageing: a review. Int J Cosmet Sci. 2008;30:87–95. doi: 10.1111/j.1468-2494.2007.00415.x 18377617

[3] Khavkin J, Ellis DAF. Ageing skin: histology, physiology, and pathology. Facial Plast Surg Clin North Am. 2011;19:229–234. doi: 10.1016/j.fsc.2011.04.003 21763983

[4] Perkins SW, Sandel HD 4th. Anatomic considerations, analysis, and the ageing process of the perioral region. Facial Plast Surg Clin North Am. 2007;15:403–407. doi: 10.1016/j.fsc.2007.08.006 18005880

[5] Hess U, Adams RB Jr, Simard A, Stevenson MT, Kleck RE. Smiling and sad wrinkles: age-related changes in the face and the perception of emotions and intentions. J Exp Soc Psychol. 2012;48:1377–1380. doi: 10.1016/j.jesp.2012.05.018 23144501 PMC3491992

[6] Klein AW . In search of the perfect lip: 2005. Dermatol Surg. 2005;31:1599–1603. doi: 10.2310/6350.2005.31247 16416644

[7] Popenko NA, Tripathi PB, Devcic Z, Karimi K, Osann K, Wong BJF. A quantitative approach to determining the ideal female lip aesthetic and its effect on facial attractiveness. JAMA Facial Plast Surg. 2017;19:261–267. doi: 10.1001/jamafacial.2016.2049 28208179 PMC5543334

[8] The Aesthetic Society . Aesthetic Plastic Surgery National Databank Statistics 2020-2021. Aesthet Surg J. 2022;42:1–18. doi: 10.1093/asj/sjac116 35730469

[9] Wu GT, Kam J, Bloom JD. Hyaluronic acid basics and rheology. Facial Plast Surg Clin North Am. 2022;30:301–308. doi: 10.1016/j.fsc.2022.03.004 35934432

[10] Bogdan Allemann I, Baumann L. Hyaluronic acid gel (Juvéderm) preparations in the treatment of facial wrinkles and folds. Clin Interv Ageing. 2008;3:629–634. doi: 10.2147/cia.s3118 PMC2682392 19281055

[11] Carruthers A, Carruthers J, Hardas B, et al A validated lip fullness grading scale. Dermatol Surg. 2008;34:S161–S166. doi: 10.1111/j.1524-4725.2008.34365.x 19021674

[12] Kane MAC, Lorenc ZP, Lin X, Smith SR. Validation of a lip fullness scale for assessment of lip augmentation [published correction appears in Plast Reconstr Surg. 2012 Jul;130(1):262]. Plast Reconstr Surg. 2012;129:822e–828e. doi: 10.1097/PRS.0b013e31824a2df0 22544112

[13] Werschler WP, Fagien S, Thomas J, Paradkar-Mitragotri D, Rotunda A, Beddingfield FC 3rd. Development and validation of a photographic scale for assessment of lip fullness. Aesthet Surg J. 2015;35:294–307. doi: 10.1093/asj/sju025 25805282 PMC4615891

[14] Lemperle G, Holmes RE, Cohen SR, Lemperle SM. A classification of facial wrinkles. Plast Reconstr Surg. 2001;108:1735–1752. doi: 10.1097/00006534-200111000-00048 11711957

[15] Tezel A, Fredrickson GH. The science of hyaluronic acid dermal fillers [published correction appears in J Cosmet Laser Ther. 2014 Jan;16(1):45]. J Cosmet Laser Ther. 2008;10:35–42. doi: 10.1080/14764170701774901 18330796

[16] Carruthers J, Cohen SR, Joseph JH, Narins RS, Rubin M. The science and art of dermal fillers for soft-tissue augmentation. J Drugs Dermatol. 2009;8:335–350. 19363852

[17] Lheritier C, Converset S, Rzany BJ, Cartier H, Ascher B. Efficacy of a new hyaluronic acid dermal filler on nasolabial folds correction: a prospective, comparative, double-blinded clinical trial. Dermatol Surg. 2024;50:746–751. doi: 10.1097/DSS.0000000000004207 38713883 PMC11288387

[18] Raspaldo H, Chantrey J, Belhaouari L, et al Lip and perioral enhancement: a 12-month prospective, randomized, controlled study. J Drugs Dermatol. 2015;14:1444–1452. 26659938

[19] Geronemus RG, Bank DE, Hardas B, Shamban A, Weichman BM, Murphy DK. Safety and effectiveness of VYC-15L, a hyaluronic acid filler for lip and perioral enhancement: one-year results from a randomized, controlled study. Dermatol Surg. 2017;43:396–404. doi: 10.1097/DSS.0000000000001035 28157728

[20] Taylor SC, Downie JB, Shamban A, et al Lip and perioral enhancement with hyaluronic acid dermal fillers in individuals with skin of color. Dermatol Surg. 2019;45:959–967. doi: 10.1097/DSS.0000000000001842 30789512

[21] Trinh LN, Grond SE, Gupta A. Dermal fillers for tear trough rejuvenation: a systematic review. Facial Plast Surg. 2022;38:228–239. doi: 10.1055/s-0041-1731348 34192769

[22] Brandt F, Bank D, Cross SL, Weiss R. A lidocaine-containing formulation of large-gel particle hyaluronic acid alleviates pain. Dermatol Surg. 2010;36:1876–1885. doi: 10.1111/j.1524-4725.2010.01777.x 20969665

[23] Royo de la Torre J, Moreno-Moraga J, Isarría MJ, et al The evaluation of hyaluronic acid, with and without lidocaine, in the filling of nasolabial folds as measured by ultrastructural changes and pain management. J Drugs Dermatol. 2013;12:e46–e52. 23545926

[24] Cartier H, Trevidic P, Rzany B, et al Perioral rejuvenation with a range of customized hyaluronic acid fillers: efficacy and safety over six months with a specific focus on the lips. J Drugs Dermatol. 2012;11:s17–s26. 22497040

[25] Dayan S, Bruce S, Kilmer S, et al Safety and effectiveness of the hyaluronic acid filler, HYC-24L, for lip and perioral augmentation. Dermatol Surg. 2015;41:S293–S301. doi: 10.1097/DSS.0000000000000540 26618456

[26] Czumbel LM, Farkasdi S, Gede N, et al Hyaluronic acid is an effective dermal filler for lip augmentation: a meta-analysis. Front Surg. 2021;8:681028. doi: 10.3389/fsurg.2021.681028 34422892 PMC8377277

[27] Monheit G, Beer K, Hardas B, et al Safety and effectiveness of the hyaluronic acid dermal filler VYC-17.5L for nasolabial folds: results of a randomized, controlled study. Dermatol Surg. 2018;44:670–678. doi: 10.1097/DSS.0000000000001529 29701621 PMC6221389

