# Supplementary material for: Lip Enhancement and Perioral Area Correction With New Hyaluronic Acid Dermal Fillers: A Prospective Safety Study
Source: Aesthet Surg J Open Forum. 2025 Aug 29;7:ojaf101. doi: 10.1093/asjof/ojaf101 (PMC12448403; doi:10.1093/asjof/ojaf101)
Supplement: ojaf101_Supplementary_Data [file ojaf101_supplementary_data.docx]

**Supplementary Table 1.**  5-point Lip Scale

| **Class** | **Description** |
| --- | --- |
| **0** | Very thin |
| **1** | Thin |
| **2** | Moderate/Medium |
| **3** | Pronounced / Voluminous |
| **4** | Plump |

**Supplementary Table 2.** Lemperle Rating Scale (LRS)

| **Class** | **Description** |
| --- | --- |
| **0** | No wrinkles |
| **1** | Just perceptible wrinkle |
| **2** | Shallow wrinkles |
| **3** | Moderately deep wrinkle |
| **4** | Deep wrinkle, well-defined edges |
| **5** | Very deep wrinkle, redundant fold |

**Supplementary Table 3.** Global Aesthetic Improvement Scale (GAIS)

|  | **Rating** | **Description** |
| --- | --- | --- |
| 1 | Very much improved | Optimal cosmetic result from injectable in this subject |
| 2 | Much improved | Marked improvement in appearance from the initial condition, but not completely optimal for this subject; A touch-up would slightly improve result |
| 3 | Improved | Obvious improvement in appearance from the initial condition, but a touch-up or retreatment is indicated |
| 4 | No change | The appearance is essentially the same as the original condition |
| 5 | Worse | The appearance is worse than the original condition |
